# A Regulatory Role for NBS1 in Strand-Specific Mutagenesis during Somatic Hypermutation

**DOI:** 10.1371/journal.pone.0002482

**Published:** 2008-06-25

**Authors:** Likun Du, Deborah K. Dunn-Walters, Krystyna H. Chrzanowska, Tanja Stankovic, Ashwin Kotnis, Xin Li, Jiayi Lu, Gösta Eggertsen, Claire Brittain, Sergey W. Popov, Andrew R. Gennery, A. Malcolm R. Taylor, Qiang Pan-Hammarström

**Affiliations:** 1 Division of Clinical Immunology, Department of Laboratory Medicine, Karolinska Institutet at Karolinska University Hospital Huddinge, Stockholm, Sweden; 2 Department of Immunobiology, King's College London School of Medicine, Guy's Hospital, London, United Kingdom; 3 Department of Medical Genetics, the Children's Memorial Health Institute, Warsaw, Poland; 4 The University of Birmingham CRC Institute for Cancer Studies, the Medical School Edghaston, Birmingham, United Kingdom; 5 Division of Clinical Chemistry, Department of Laboratory Medicine, Karolinska Institutet at Karolinska University Hospital Huddinge, Stockholm, Sweden; 6 Department of Pediatric Immunology, Newcastle General Hospital, Newcastle, United Kingdom; Oklahoma Medical Research Foundation, United States of America

## Abstract

Activation-induced cytidine deaminase (AID) is believed to initiate somatic hypermutation (SHM) by deamination of deoxycytidines to deoxyuridines within the immunoglobulin variable regions genes. The deaminated bases can subsequently be replicated over, processed by base excision repair or mismatch repair, leading to introduction of different types of point mutations (G/C transitions, G/C transversions and A/T mutations). It is evident that the base excision repair pathway is largely dependent on uracil-DNA glycosylase (UNG) through its uracil excision activity. It is not known, however, which endonuclease acts in the step immediately downstream of UNG, i.e. that cleaves at the abasic sites generated by the latter. Two candidates have been proposed, an apurinic/apyrimidinic endonuclease (APE) and the Mre11-Rad50-NBS1 complex. The latter is intriguing as this might explain how the mutagenic pathway is primed during SHM. We have investigated the latter possibility by studying the *in vivo* SHM pattern in B cells from ataxia-telangiectasia-like disorder (Mre11 deficient) and Nijmegen breakage syndrome (NBS1 deficient) patients. Our results show that, although the pattern of mutations in the variable heavy chain (V_H_) genes was altered in NBS1 deficient patients, with a significantly increased number of G (but not C) transversions occurring in the SHM and/or AID targeting hotspots, the general pattern of mutations in the V_H_ genes in Mre11 deficient patients was only slightly altered, with an increased frequency of A to C transversions. The Mre11-Rad50-NBS1 complex is thus unlikely to be the major nuclease involved in cleavage of the abasic sites during SHM, whereas NBS1 might have a specific role in regulating the strand-biased repair during phase Ib mutagenesis.

## Introduction

Mammalian organisms require two types of DNA recombination in order to produce functional antibody encoding genes. The first, V(D)J recombination, mediates assembly of the variable domains of the immunoglobulin (Ig) heavy and light chains in pre-B cells. In the second, class switch recombination (CSR), the constant region gene of the μ heavy chain (Cμ) is replaced by a downstream C_H_ gene, resulting in a change from IgM to IgG, IgE, or IgA production. Both types of recombination require double-strand breaks (DSBs) as intermediates, and mechanisms for genomic stability, especially the non-homologous end-joining (NHEJ) pathway(s), are utilized in these processes [1]–[3].

Somatic hypermutation (SHM), where point mutations are introduced at a high rate into the Ig variable (V) genes, is another important process that shapes the Ig repertoire. SHM and CSR can occur independently in the germinal center but are both initiated by a single B cell-specific factor, activation-induced cytidine deaminase (AID) [4], probably through deamination of deoxycytidine (dC) to deoxyuridine (dU) residues within the Ig locus [5], [6]. The initial lesion in the V genes and switch (S) regions are, however, resolved differently, as DSBs seem not to be prominent intermediates in SHM and one of the core NHEJ factors, DNA-PKcs, is dispensable for this process [7]. Conversely, single strand breaks (SSBs) or single strand nicks appear to be associated with SHM [8]–[10]. At least two pathways, base excision repair and mismatch repair, have been implicated in processing of the dU: deoxyguanosine (dG) lesions in the V regions during SHM [10]. However, the mechanism by which they result in a mutagenic, rather than a faithful repair remains elusive. Furthermore, these pathways are also involved in CSR and the way in which they are regulated and coordinated to mediate SHM or CSR is still not well understood.

It is evident that the base excision repair pathway operating in SHM and CSR is largely dependent on uracil-DNA glycosylase (UNG) [5], [11], through its uracil excision activity [12]. It is not known, however, which endonuclease acts in the subsequent step, i.e. recognizes the abasic sites generated by UNG and converts them to SSBs. The obvious candidate is an apurinic/apyrimidinic endonuclease (APE or APEX), which functions in the conventional base excision repair. In mammalian cells, APE1 is the major APE [13], [14] and it is essential for early embryonic development in mice [15]. A second APE, APE2, has also been identified [16] and mice with a targeted inactivation of the *APEX2* gene show thymic atrophy and reduced number of B cells [17], suggesting that APE2 may have unique functional properties in the lymphoid system that cannot be compensated by APE1. Recently, Guikema et al have suggested that both APEs are involved in CSR, based on observation that CSR and DSBs in Sμ are moderately reduced in mice deficient in APE2 or haploinsufficient for APE1 [18]. A potential role for these factors in SHM has, however, not been identified.

An alternative candidate, the Mre11-Rad50-NBS1 (MRN) complex, has recently been proposed to compete with APE1 for cleavage of abasic sites and to direct a mutagenic pathway [19]. This multi-subunit nuclease is required for telomere maintenance, cell cycle checkpoint signaling, DNA replication, meiotic recombination and repair of DSBs by homologous recombination and/or NHEJ [20], [21]. In mice, disruption of any subunit of the MRN complex results in embryonic lethality [22]–[24]. In humans, mutations in the genes encoding Mre11 and NBS1 result in two rare chromosomal instability syndromes: ataxia-telangiectasia-like disorder (ATLD) and Nijmegen breakage syndrome (NBS) [25], [26]. By studying B cells from these patients, we have previously shown that CSR is less efficient when either Mre11 or NBS1 is deficient [27], [28]. In addition, skewed mutation patterns at the switch recombination junctions have been observed in both ATLD and NBS patients, suggesting that the MRN complex may be involved in repair/editing of DNA breaks/ends in the S regions [27], [28]. The requirement of NBS1 for efficient CSR has also been demonstrated by analyzing NBS1-deficient mouse B cells using a conditional knockout strategy [29], [30]. In addition to its role in CSR, the MRN complex has recently been implicated in SHM, as hypermutation in the human B cell line Ramos is accelerated by ectopic expression of NBS1 [31]. Furthermore, Mre11, rather than APE1, has been shown to be associated with rearranged Ig genes in hypermutating B cells [19]. Moreover, in an *in vitro* assay, Mre11/Rad50 cleaves at abasic sites within single-stranded regions of DNA [19]. To test whether the MRN complex is indeed involved in SHM and the possibility that it might act downstream of UNG, we analyzed the *in vivo* SHM patterns in the Ig locus in cells from ATLD (Mre11 deficient) and NBS (NBS1 deficient) patients.

## Results

### SHM pattern in V_H_3-23-Cγ transcripts in ATLD patients

**Figure 1:**
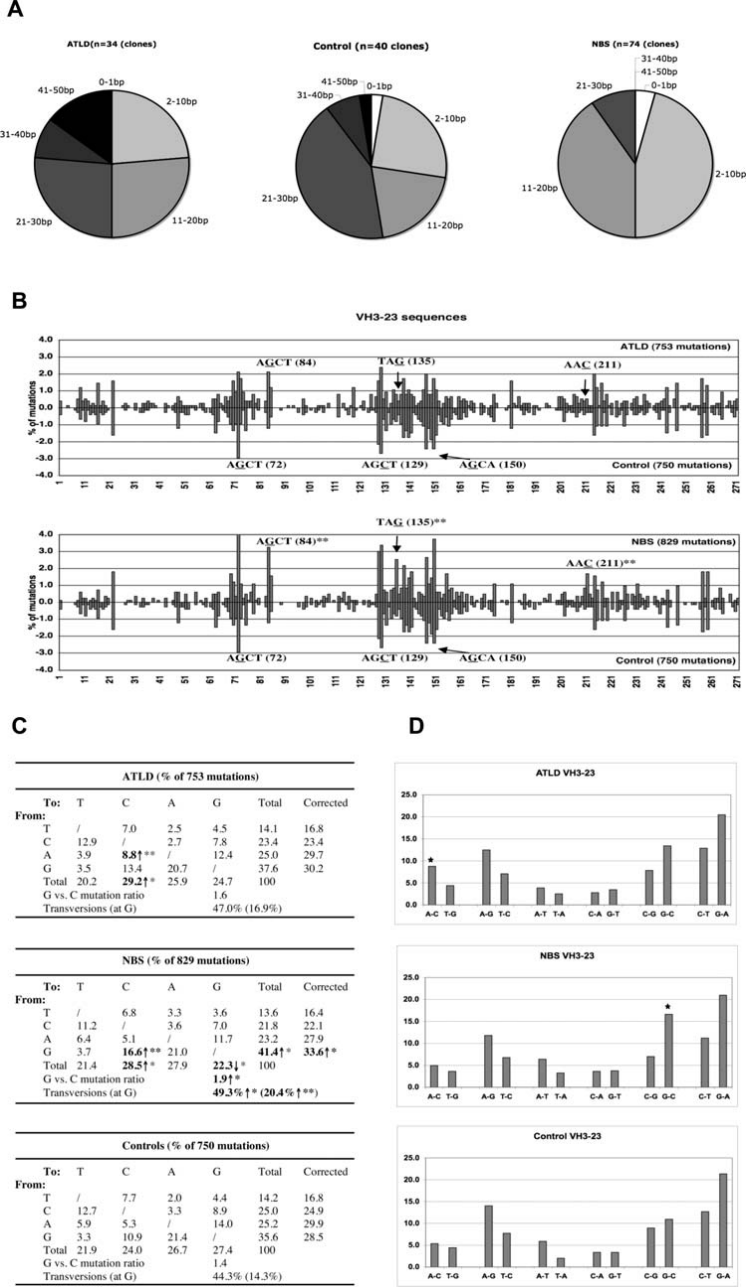
Pattern of mutations introduced in the V_H_3-23-Cγ transcripts. A). Pie charts demonstrate the proportion of clones carrying the indicated number of mutations. B). Distribution of mutations in V_H_3-23 in ATLD or NBS patients (above the zero line) and in controls (below the zero line). Nucleotides are numbered from the third base of codon 10 of the V_H_3-23 coding region. The percentage of mutations at each position (number of mutations at each position / total number of mutation) is plotted and the major hotspots in controls are highlighted. Several positions that show significantly more mutations in the NBS patients are also indicated. **p<0.01. C). Nature of the base substitutions in the V_H_3-23 genes. The germline sequence used for comparison contains 21.4% A, 31.7% G, 21.4% T and 25.5% C. Correction: values presented in the column were corrected to represent sequences with equal amounts of the four nucleotides. Statistical analysis was performed using χ^2^ test. Numbers that are significantly different from controls are bolded. *p<0.05; **p<0.01. ↑, significantly increased; ↓, significantly decreased. D). Percent distribution of each type of nucleotide substitution. Individual substitutions are paired together with their complementary mutations. Mutations in ATLD or NBS V_H_3-23 sequences were compared with the control V_H_3-23 transcripts. Stars indicate that the proportion of a given substitution when it is significantly different in patients as compared to the controls.

RNA was prepared from peripheral blood mononuclear cells (PBMC) from 4 ATLD patients. In total, 34 distinct V_H_3-23-Cγ clones were generated and all clones were mutated (2–50 bp substitutions/clone, average 22.1 bp) (Fig. 1A). Overall, the frequency of mutations in the V_H_3-23 genes derived from ATLD patients varied from 5.9% to 8.9%, which is similar to that found in controls (3.4%-9.3%, average 6.9%; Table 1). The ratio of replacement vs. silent (R/S) mutations in the CDRs (CDR1-2) and FR (FR1-3) was also similar in the patient and control groups.

**Table 1:** Mutations in V_H_3-23-Cγ transcripts from PMBC from NBS and ATLD patients.

			Clones			Mutations in V region		Clustering ratio	R/S	
	Age (yr)	Germline mutation	All	Distinct	Unmutated	Total	% bp	CDR/FR^a^	CDRs	FRs
**ATLD patients**										
ATLD1	36	1897T>C (ho)	19	13	0	270	7.7	1.4	3.4	1.9
ATLD2	29	1897T>C (ho)	14	9	0	216	8.9	1.3	5.1	2.2
ATLD3	23	350G>A (he), 1714T>C (he)	14	11	0	251	8.4	1.2	5.6	2.0
ATLD4	26	350G>A (he), 1714T>C (he)	1	1	0	16	5.9	2.0	–^b^	–^ b^
**Total (n = 4)**	**23–36**		**48**	**34**	**0**	**753**	**8.2**	**1.3**	**4.5**	**2.0**
							(5.9–8.9)	(1.7–2.0)	(3.4–5.6)	(1.9–2.2)
**NBS patients**										
NBS1	8	657–661del5 (ho)	14	12	0	150	4.6	2.2	6.7	1.8
NBS2	10	657–661del5 (ho)	14	12	0	169	5.2	1.5	5.1	2.4
NBS3	8	657–661del5 (ho)	16	13	1	124	3.5	1.7	2.3	1.8
NBS4	6	657–661del5 (ho)	17	14	0	91	2.4	1.9	18.5	2.7
NBS5	16	657–661del5 (ho)	16	14	0	177	4.7	1.6	3.1	2.2
NBS6	2	1089C>A (ho)	18	9	2	118	4.8	1.2	36	1.8
**Total (n = 6)**	**2–16**		**95**	**74**	**3**	**829**	**4.1**	**1.7**	**5.0**	**2.1**
							(2.4–5.2)	(1.2–2.2)	(2.3–36)	(1.8–2.7)
**Controls^c^**										
**Total (n = 5)**	**14–30**		**41**	**40**	**1**	**750**	**6.9**	**1.8**	**6.2**	**1.8**
							(3.4–9.3)	(1.3–3.2)	(4.5–14)	(1.2–2.2)
**Controls II**										
**Total (n = 7)**	**1–8**		**114**	**96**	**12**	**808**	**3.1**	**1.5**	**5.2**	**2.3**
							(1.9–4.6)	(1.1–2.4)	(2.5–10)	(1.5–3.1)

a) CDR, complementary determining region; FR, framework region.

b) Too few mutations to give a reliable ratio.

c) The V_H_3-23 sequences from controls have been described previously [60].

The distribution of mutations in the V_H_3-23 sequence in ATLD patients was similar to that observed in controls (Fig. 1B) and there was no significant difference in targeting of any of the 271 positions between patients and controls. Furthermore, no major differences were found in the nature of base pair substitutions between ATLD patients (753 mutations) and normal controls (750 mutations), except that there was a small, albeit significant, increase of A to C transversions in the ATLD patients (8.8 vs. 5.3% in controls, χ^2^ test, *p*<0.01).

To search for a potential cause for the increased frequency of A to C transversions in ATLD patients, we analyzed the frequency of mutations in each of the 64 possible trinucleotide combinations. Significantly increased targeting of the A residues was observed in AGA, CAA and CGA, whereas significantly decreased targeting of A was noticed in CTA (Table S1). These trinucleotides are neither related to the AID targeting (WRC/GYW) (W, A or T; R, A or G; Y, C or T) motifs, nor the SHM hotspot motifs (RGYW/WRCY), and no difference in targeting of the A residues within these motifs could be observed between ATLD patients and controls (Table 2 and Table S3). Two of these trinucleotides, CAA and CTA, are related to the previously described SHM hotspots (WA/TW) for A/T mutations [32]. There was indeed a significantly decreased targeting of the A residues in the TA motifs (9.2% vs. 13.3%, χ^2^ test, *p*<0.05) and a small, but not significant, increase in targeting of the AA motifs (7.2% vs. 5.3%) in ATLD patients. The frequency of A to C transversions was, however, increased in both the TA and AA motifs (35% vs. 21% of total A mutations within the TA and 44% vs. 23% within the AA motifs), and also outside these motifs (34% vs. 22%). We thus conclude that the frequency and distribution of mutations in the V_H_ regions are normal in ATLD patients. The general pattern of base substitutions was slightly altered, with an increased frequency of A to C transversions. This alteration was, however, not associated with differential targeting of any of the known SHM hotspots.

**Table 2:** Number of mutations at AID targeting hotspots in V_H_3-23 and J_H_4 intronic sequences

		V_H_3-23 sequences			J_H_4 intronic sequences	
		ATLD	NBS	Control	NBS	Control
		(753 mutations)	(829 mutations)	(750 mutations)	(386 mutations)	(797 mutations)
**WRC**						
Position	1-A	42 (5.6%)	34 (4.1%)	40 (5.3%)	30 (7.8%)	42 (5.3%)
	-T	14 (1.9%)	**23** (2.8%)**	7 (0.9%)	11 (2.8%)	17 (2.1%)
Position	2-A	25 (3.3%)	40 (4.8%)	27 (3.6%)	5 (1.3%)	18 (2.3%)
	-G	83 (11.0%)	**151** (18.2%)***	88 (11.7%)	50 (13.0%)	78 (9.8%)
Position	3-C	110 (14.6%)	126 (15.2%)	112 (14.9%)	62 (16.1%)	123 (15.4%)
Total		275 (36.5%)	**375** (45.2%)***	277 (36.9%)	**158** (40.9%)*	278 (34.8%)
**GYW**						
Position	1-G	170 (22.6%)	**243** (29.3%)**	170 (22.7%)	**79** (20.5%)**	107 (13.4%)
Position	2-C	82 (10.9%)	84 (10.1%)	78 (10.4%)	31 (8.0%)	70 (8.8%)
	-T	20 (2.7%)	21 (2.5%)	26 (3.5%)	2 (0.5%)	7 (0.9%)
Position	3-A	59 (7.8%)	60 (7.2%)	71 (9.5%)	27 (7.0%)	31 (3.9%)
	-T	19 (2.5%)	15 (1.8%)	12 (1.6%)	8 (2.1%)	23 (2.9%)
Total		350 (46.5%)	423 (51.0%)	357 (47.6%)	**147** (38.1%)*	238 (29.9%)
**GYW and WRC (duplicates excluded)**						
G mutations		186 (24.7%)	**253** (30.5%)**	180 (24.0%)	**87** (22.5%)**	118 (14.8%)
**All sequences**						
G mutations		383 (37.6%)	**343** (41.4%)*	267 (35.6%)	**147** (38.1%)**	230 (28.9%)

### SHM pattern in V_H_3-23-Cγ transcripts in NBS patients

RNA samples from 6 NBS patients (NBS1-6) were subsequently analyzed. In total, 74 distinct V_H_3-23-Cγ clones were obtained and a majority of these clones were mutated (2–28 bp substitutions/clone, average 11.2 bp; Fig. 1A). The frequency of mutations in the V_H_3-23 genes derived from NBS patients varied from 2.4% to 5.2%, with an average of 4.1%, which was lower than in the controls (3.4%–9.3%, average 6.9%, Table 1). The ratio of R/S mutations in the CDRs and FRs was within the normal range in most patients, except in NBS6, where nearly all the observed mutations in the CDR regions were replacements (Table 1).

The distribution of mutations observed in NBS patients was largely similar to that found in normal controls, with major hot spots of mutation at previously described RGYW/WRYC motifs (AGCT at position 72 and 129 and AGCA at position 150; Fig. 1B). However, a slight increase, albeit significant, number of mutations was observed at these three hotspots in NBS patients (11.2% vs. 8.0% of total mutations, χ^2^ test, *p*<0.05). In addition, we also found a significantly increased targeting at a few positions that are normally not highly mutated in the V_H_ genes (AGCT, TAG, and AAC at positions 84, 135 and 211; Fig. 1B). Furthermore, the general pattern of base substitution in the NBS patients (829 mutations) was somewhat different from that in controls (750 mutations), with significantly more transversions (49.3% vs. 44.3%, χ^2^ test, *p*<0.05) and substitutions occurring at G residues (41.4% vs. 35.6%, χ^2^ test, *p*<0.05; Fig. 1C). These were mainly due to an increased frequency of G transversions (20.4% vs. 14.3%, χ^2^ test, *p*<0.01), especially G to C transversions, in the NBS patients (16.6% vs. 10.9%, χ^2^ test, *p*<0.01; Fig. 1C and 1D). There seems to be a gain of strand polarity at G/C pairs in the V region in NBS patients, where the G residues on the top (non-transcribed, coding) strand were preferred targets for mutations (G/C ratio 1.9 vs. 1.4 in controls; χ^2^ test, *p*<0.05). The strand preference at A/T pairs was, however, not affected (A/T ratio 1.7 vs. 1.8 in controls).

To search for a potential cause for the increased targeting of G residues in NBS patients, we first analyzed the sequences surrounding individual mutated bases (−2 to +2) in the V_H_3-23 genes. The preferred target motif for G mutation was TAGYW for both patients and controls, confirming the known hotspots for G mutations (RGYW/WRCY). We subsequently performed a similar analysis as described above for the targeting of trinucleotides (Table S2). Significantly increased targeting of the G residues was observed in AGC, GCA, GCT and TAG in the V_H_3-23 genes in the NBS patients.

**Figure 2:**
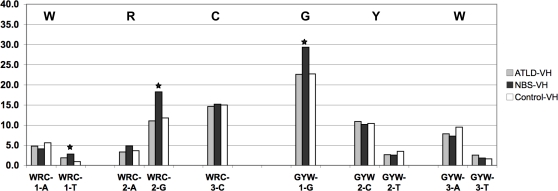
Frequency of mutations within the WRC/GYW motifs in V_H_3-23 sequences. Stars indicate that the frequency of mutations at a given position when it is significantly different in patients as compared to controls.

As the first three trinucleotides are all related to the AID targeting (WRC/GYW) and/or SHM hotspot motifs for G/C mutations (RGYW/WRCY), we subsequently analyzed the frequency of G mutations within, or outside, these motifs. A significantly higher number of G mutations was indeed observed in the WRC or GYW motifs in NBS patients (Fig. 2 and Table 2), whereas G residues outside these motifs were targeted equally (11.3% vs. 11.1% of total mutations in controls). The G mutations occurring in the overlapping WRC and GYW motifs (WGCW) were, however, counted twice when analyzing the WRC and GYW motifs separately. When this was taken into account, an increased targeting of the G nucleotides within the GYW motifs (including those within the overlapping motifs) could explain most of the difference between NBS patients and controls in terms of targeting of the G residues (Table 2), and most of the G mutations within the WRC motifs were actually located within the palindromic sequence WGCW. Furthermore, within the GYW motifs, the proportion of G to C transversions was significantly higher in NBS patients (38.3% vs. 27.1% of the total G mutations within these motifs, χ^2^ test, *p*<0.05). Similar results were obtained when analyzing the RGYW/WRCY motifs, where an increased targeting of the G nucleotides within the RGYW motifs (including the “dual” motif AGCT) could explain most of the difference between NBS patients and controls (Table S3).

It is interesting to note that while, on the top strand, the G residues at the first position of the GYW or the second position of the RGYW motifs were significantly more mutated in NBS patients, no alteration in the rate of mutations was noted in the corresponding C residues at the reverse complementary motifs (WRC or WRCY; Table 2, Fig. 2 and Table S3). The C residues within the AID hotspot in the bottom strand (3′-CRW-5′) seemed to be more mutated in NBS patients, while those in the top strand (5′WRC-3′) were not affected. The altered pattern of base pair substitutions in the V_H_ genes in NBS patients may thus arise from asymmetric targeting of AID, or linked repair steps.

As the NBS patients are younger (2–16 years old) than the controls (14–30 years old), we subsequently studied the frequency and pattern of mutations in the V_H_3-23 genes from an additional group of controls, aged 1–8 years. The frequency of mutations in the V_H_3-23 genes derived from the second group of controls varied from 1.9% to 4.6%, with an average of 3.1%, which is lower as compared to the first group of controls (average 6.9%), but comparable with the NBS patients (average 4.1%) (Table 1). The patterns of base pair substitutions in the V_H_3-23 genes derived from the two groups of controls were largely similar. When the NBS patients were compared with the second group of controls, we could confirm that there were significantly more G mutations (41.4% vs. 35.9%, χ^2^ test, *p*<0.05), especially G to C transversions (16.6% vs. 12.0%, χ^2^ test, *p*<0.01), in the patient sequences (Table S4). Furthermore, the G residues at the first position of GYW (29.3% vs. 23.0%, χ^2^ test, *p*<0.01) or the second position of RGYW (22.9% vs. 18.4%, χ^2^ test, *p*<0.05) motifs were significantly more targeted in the patients whereas targeting of the corresponding C residues in the WRC or WRCY motifs was not affected. We thus conclude that the frequency of mutations is still within the normal range when NBS patients are compared to controls with a similar age. The nature of base pair substitutions in the V_H_ genes in NBS patients is however altered, and these changes are not associated with the younger age of patients.

### Mutation pattern in J_H_4 intronic regions in NBS patients

The number of mutations (n = 7) obtained from nonproductive NBS V_H_3-23 rearrangements was not sufficient for further analysis. To exclude the possibility that the altered mutation pattern in NBS cells was a result of selection bias introduced by studying the expressed V_H_ genes only, we analyzed the J_H_4 intronic sequences in 7 NBS (NBS6-12) patients and 10 controls. We were able to obtain DNA samples from CD27^+^ PBMC in one patient (NBS6) and nine controls, where fresh blood samples were available. For the remaining patients and controls, DNA samples were obtained from PBMC. More than 1750 clones were screened and 652 clones were fully sequenced. After removing clones with the same V(D)J junctions or J_H_4 intronic sequences, 279 and 175 distinct clones were obtained from the NBS patients and controls respectively. There was a marked variation in mutation frequency both within the patient group (0.02%–0.70%) and controls (0.00%–2.21%), although, in general, the rate of mutations was lower in the NBS patients (average 0.45% vs. 1.48% in controls). However, taking into account that CD27^+^ cells were enriched in the controls, the frequency of mutations in NBS patients was still comparable to controls.

**Figure 3:**
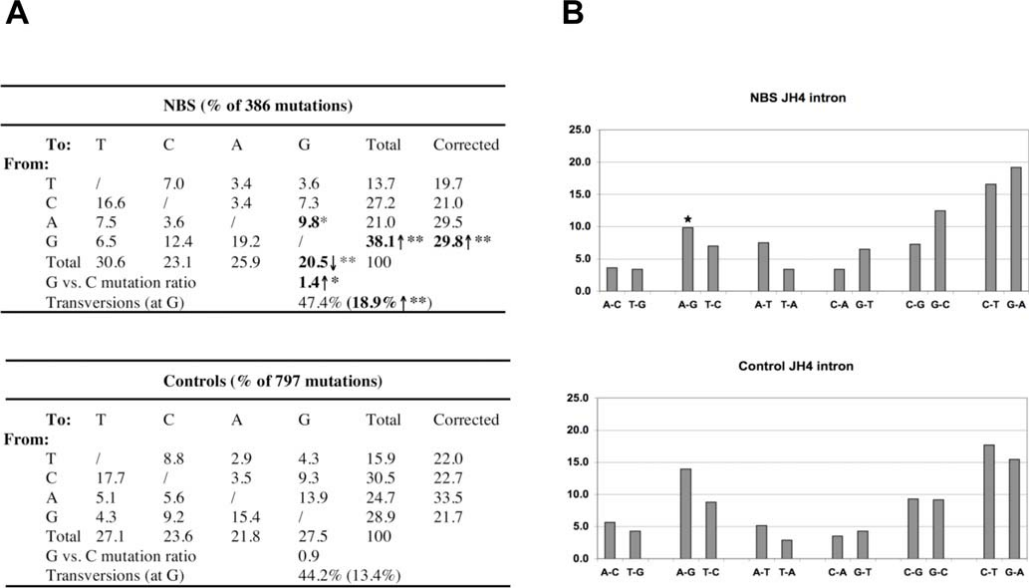
Pattern of mutations introduced in the J_H_4 intronic sequences. A). Nature of the base substitutions in the J_H_4 intronic genes. The germline sequence used for comparison contains 17.9% A, 32.1% G, 17.5% T and 32.5% C. Correction: values presented in the column were corrected to represent sequences with equal amounts of the four nucleotides. Statistical analysis was performed using χ^2^ test. Numbers that are significantly different from controls are bolded. *p<0.05; **p<0.01. B). Percent distribution of each type of nucleotide substitution. Stars indicate that the proportion of a given substitution is significantly different in NBS patients as compared to that in controls.

The pattern of base substitutions in the J_H_4 intronic regions derived from NBS patients (386 mutations) was again different from that in controls (797 mutations) and we confirmed that, as in the V_H_3-23 transcripts, there were significantly more transversions occurring at G residues (18.9% vs. 13.4%, χ^2^ test, *p*<0.01) and G mutations altogether (38.1% vs. 28.9%, χ^2^ test, *p*<0.01). However, unlike in the V_H_3-23 transcripts, increased numbers of all types of G mutations (G to C, G to T and G to A) were observed, although none of the individual counts reached a statistically significant degree (Fig. 3A; χ^2^ test, *p* = 0.08, *p* = 0.10 and *p* = 0.11 respectively). In addition, there were also significantly fewer A to G substitutions in the NBS J_H_4 intronic sequences as compared to those from controls (Fig. 3). The preference for G, rather than C, residues on the top strand, a gain of strand polarity at G/C pairs, was again notable in the NBS patient samples (G/C ratio 1.4 vs. 0.9 in controls; χ^2^ test, *p*<0.05), and as in the V_H_ sequences, the strand preference for A/T pairs was not affected (A/T ratio 1.5 vs. 1.6 in controls). Furthermore, we could see a significant trend where the G residues in the first position of the GYW motifs (Table 2) or the second position of RGYW motifs (Table S3) were more mutated in the NBS patients, whereas no difference in targeting of the corresponding C residues in the reverse complementary motifs (WRC or WRCY) was observed (Table 2 and S3). Thus, in NBS patients, the mutation pattern in the J_H_4 intronic sequences largely recaptures the features in the V_H_ regions, where an increased number of the G, but not C, mutations are observed in SHM and/or AID hotspots.

### Characteristics of the CDR3 in B cells from ATLD and NBS patients

Diversity in the CDR3 region in clones derived from the ATLD and NBS patients was also analyzed. The CDR3 contains contributions from the V_H_, D, and J_H_ gene segments and nucleotides added by TDT. The majority of analyzable clones were in-frame rearrangements (27 of 30 ATLD clones and 69 of 72 NBS clones). The average length of the CDR3 in the ATLD and NBS clones was similar to those from controls (Table S5). The length of the N regions and the number of P nucleotides in the ATLD clones were comparable to the controls. The average length of the N2 region (D-J_H_ junctions) was, however, significantly shorter in the NBS clones as compared to controls (Student's *t* test, *p*<0.05; Table S5) but no significant difference in the length of the N1 regions (V_H_-D junction) or the frequency of P nucleotides was observed between NBS patients and controls. We thus conclude that the pattern of V(D)J coding joints are largely normal in ATLD and NBS patients.

## Discussion

It has previously been suggested that the MRN complex participates in all three AID-initiated processes that diversify the Ig genes; CSR, SHM and gene conversion. Support for the latter two mechanisms is, however, indirect, and based on experiments using ectopic expression of NBS1 in B cell lines [31]. “Null” mutations in the gene encoding any subunit of the MRN complex result in embryonic lethality in mice [22]–[24], suggesting that this complex is essential in mammals. Cells from ATLD or NBS patients, who carry “hypomorphic” mutations in the *Mre11* or *NBN* gene, have been used as an alternative system to analyze the functional properties of the MRN complex in DNA damage signaling and DNA repair, as these cells have either no, or markedly reduced levels of the wild type proteins [33], [34]. In this study, we therefore assessed the role of the MRN complex in SHM, by analyzing the mutational spectrum generated *in vivo* in V_H_ genes in cells from ATLD and NBS patients. Largely normal frequencies of mutations in both groups of patients suggest that the complex is not essential for SHM.

**Figure 4:**
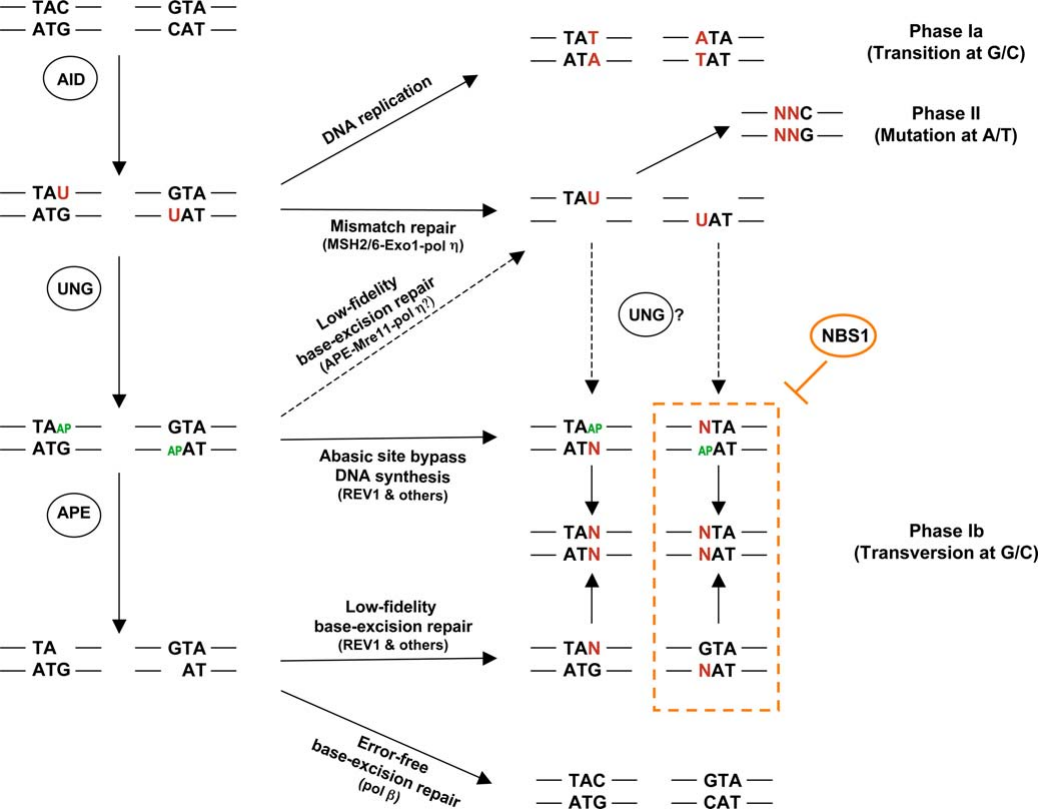
Hypothetical model for strand-biased mutagenic repair during SHM. AID is believed to initiate SHM through deamination of the dC residues, probably on both strands. In phase Ia, replication over dU, without repair, creates G/C transitions [5]. In phase Ib, removal of the uracil by UNG produces an abasic site that can be cleaved by an APE and subsequently repaired by low-fidelity polymerases, leading to transitions as well as transversions at G/C residues. Alternatively, mutations at G/C residues might arise if a bypass DNA polymerase inserts a base opposite to an abasic site before the lesion has been recognized by APE. The mismatch repair pathway may contribute a small fraction of phase Ib mutation although the exact mechanism is not known. During phase Ib, NBS1 promotes error-free base-excision repair and inhibits the abasic site bypass DNA synthesis or low-fidelity base excision repair, if the abasic site is located in the bottom strand. In phase II, mutations are introduced at adjacent positions, predominantly at nearby A/T pairs, mainly through a MSH2/MSH6 triggered, error-prone, patch repair process. Based on the study by Unniraman et al [62], the top-strand deaminated C residues contribute to most of the mutations at A/T residues. The low-fidelity base excision pathway, together with pol η, may contribute a small proportion of the A/T mutations and Mre11 might be involved in this minor pathway. Base alterations are shown in red. AP, abasic site.

The first two components of the MRN complex, Mre11 and Rad50, have been shown to have abasic site-lyase activity, with a preference for single stranded DNA [19]. The DNA ends produced by Mre11/Rad50 cleavage cannot directly prime new DNA synthesis by polymerase β (pol β), which carries out high-fidelity repair. Mre11 has therefore been proposed to compete with APE1 for cleavage of abasic sites and to direct a mutagenic pathway during SHM [19]. This hypothesis would predict that Mre11 is involved in the UNG dependent pathway, which is mainly responsible for generation of phase 1b mutations (G/C transversions) but, together with polymerase η (pol η), also generates a small proportion of phase II mutations (A/T mutations) [35]. In ATLD patients (Mre11 deficient), we did observe a perturbed mutation pattern, with a small increase of A to C transversions (phase II). The number of G to C transversions (phase Ib) was, however, only slightly increased and the difference did not reach statistical significance (Fig. 1C and 1D). As the UNG-pol η pathway probably only serves as a backup for the MSH2-dependent pathway in phase II mutagenesis [35], one would not expect a major change in the mutation pattern or total mutation load at A/T bases. We would, however, expect a more pronounced change in the rate of G/C transversions (decreased rather than increased) in ATLD patients. Thus, Mre11 is unlikely to be the major enzyme used to cleave the abasic sites during SHM. Nevertheless, the increased rate of A to C transversions was only observed in ATLD patients and, notably, these changes were more prominent in patients ATLD1 and ATLD2, who carry homozygous 1897 C>T (R633stop) mutations in the *Mre11* gene. The truncated Mre11 protein expressed in cells from these two patients show weaker interaction with Rad50 as compared with those from ATLD3 and ATLD4 [25]. As pol η is responsible for most of the A to G and A to T substitutions, but only about 50% of the A to C mutations [36], an increased frequency of the latter could indicate a lower activity of pol η and/or a higher activity of other polymerases. Thus, the possibility remains that there is a subtle defect in the UNG-Mre11-pol η pathway (Fig. 4). APE1 and APE2 remain the best candidates for cleavage of the abasic sites during SHM, although direct evidence for their involvement is still missing and potential participation of other, less well-characterized, AP endonucleases such as PALF (PNK and APTX-like FHA protein) also needs to be excluded [37].

The skewed mutation pattern in NBS1 deficient patients has not been observed in any mouse or human disease model. The alteration (increased G mutations) is closely associated with SHM and/or AID hotspots and causes a G over C strand bias. It is possible that, in the normal situation, NBS1 facilitates generation of mutations from the C residues on the top strand or prevents mutations of the deaminated C residues on the bottom strand. Potentially, in the presence of NBS1, the top strand (non-transcribed, coding) is more targeted by AID than the bottom strand (transcribed, non-coding), or maybe a deaminated C residue, located on the top strand, is processed differently from one located on the bottom strand (mutagenically repaired or faithfully repaired). Based on data from UNG and Msh2 double knockout mice, it has been suggested that AID deaminates the top and bottom strands equally well [5], [38]. If this is true (there is still some debate [39]), NBS1 is more likely to be involved in, or regulating, a strand-specific mutagenic repair step after the initial AID targeting.

When NBS1 was overexpressed in the human B cell line Ramos, an increased frequency of mutations in the V_H_ genes was observed [31]. The mutation spectrum was also altered, with proportionally more C than G mutations [31]. This may suggest that NBS1 facilitates generation of mutations from the C residues in the top strand. However, in our NBS patients (NBS defective), the mutation frequency in general is largely normal, and although an increased number of G mutations was observed in the GYW motifs (AID hotspots in the bottom strand), the frequency of mutations at the C residues in the top-strand hotspot (WRC) was not altered. Thus, based on our *in vivo* data, NBS1 is more likely to influence the outcome of deaminated C residues in the bottom strand (faithfully repaired or mutated), rather than to promote generation of mutations from the top strand.

How might then NBS1 be regulating the mutagenic repair in the bottom strand? One possibility is that by regulating the S phase checkpoint and interacting with the DNA replication machinery [40]–[43], NBS1 inhibits abasic site bypass DNA synthesis by certain translesion synthesis polymerases, if the abasic sites are located in the bottom strand (Fig. 4). When NBS1 is defective, these polymerases will be able to bypass the DNA lesion in the bottom strand as well, by incorporating a base opposite an abasic site. Rev1 could be one such polymerase, which inserts a C opposite an abasic site. In its absence, the C to G transversions are completely absent on the top strand, but only partially reduced on the bottom strand, suggesting that it has a preference for the top strand [44]. Thus, a loss of strand preference of Rev1 could explain the “net” increase of G to C transversions (i.e. C to G transversions on the bottom strand) in the V_H_ genes in NBS patients. Alternatively, following cleavage of the abasic site by APE, NBS1 inhibits the action of low-fidelity polymerases, if the abasic site is located on the bottom strand (Fig. 4). In addition to the above hypothesis that NBS1 is involved in, or regulating the “post-UNG” events during base-excision repair, the possibility that it interacts with the mismatch repair pathway should also be considered. The MSH2-dependent mismatch repair pathway can recognize the dU:dG lesions and it is responsible for generating small proportion of the G/C mutations, including G/C transversions [35]. Perhaps NBS1 can direct the choice of which of the strands is being removed by mismatch repair [45] (Fig. 4). Regardless of the mechanism involved, our data suggests that transversions at G/C residues (phase Ib mutations) are also generated in a strand-biased manner. NBS1 may be regulating this process by promoting error-free repair on the bottom strand.

Most of the NBS patients included in this study carry a deletion mutation (675del5), which was originally regarded as a null mutation. Later, it was shown that in addition to the predicted NBS1 p26 protein, through alternative translation, another truncated protein, NBS1 p70 is produced by the patient cells [46]. This protein lacks the N-terminal FHA and BRCT domains but can still associate with Mre11/Rad50 and is probably responsibe for the cell viability [46], [47]. As the G/C mutation pattern in ATLD patients is largely normal, the role of NBS1 in SHM is probably uncoupled from Mre11. As the level of NBS1 is also decreased in cells from the ATLD patients included in this study (ATLD1-4) [25], [48], the moderately increased rate of G to C transversions noted might be a consequence of a reduced level of NBS1, rather than deficiency of Mre11.

The mutation pattern at the CSR junctions was altered in both the NBS and ATLD patient groups [27]. In ATLD patients, there were fewer base substitutions due to transitions and, most strikingly, the substitutions that occurred most often in controls, C to T transitions, never occurred [27]. In NBS patients, base substitutions were only observed at the G/C nucleotides, with a preference for transitions [27]. These peculiar patterns of base substitutions at CSR junctions were, however, quite different from the patterns we observed in the V_H_ regions from these patients. Thus, although the MRN complex might be involved in both CSR and SHM, the underlying mechanisms could be quite different. For instance, the role of the MRN complex during CSR could be attributed to its activity in the ATM signaling pathway [49], [50], or through its interaction with the NHEJ pathway. This would, however, not explain the role of the MRN complex in SHM, as the ATM dependent signaling and the classical NHEJ pathway are critical for CSR [28], [51]–[54], but not for SHM [7], [55].

In summary, the SHM mutation pattern in the V_H_ genes in NBS patients was altered, with a significantly increased number of G transversions occurring in the SHM and/or AID targeting hotspots. The general pattern of mutations in the V_H_ genes in ATLD patients was slightly altered, with an increased frequency of A to C transversions. Mre11 is thus unlikely to be the major enzyme used to cleave abasic sites, whereas NBS1 might have an Mre11-independent role in regulating the strand-specific mutagenic repair process during SHM, although the actual mechanism remains elusive.

## Materials and Methods

### Patient material

The study included 4 ATLD patients from two independent families and the clinical details of these patients have been described previously [25]. The mutations in *Mre11* in these patients are shown in Table 1. The study also included 11 Polish NBS patients (NBS1-5, NBS7-12) and 1 Pakistani NBS patient (NBS6). The diagnosis of these patients was carried out in the respective centers in the Children's Memorial Health Institute (Poland) and the Newcastle General Hospital (UK). The truncating 5-bp deletion (657del5) in the *NBN* gene is present in a homozygous form in patients NBS1-5 and NBS7-12, whereas a homozygous nonsense mutation (1089C>A) has been identified in NBS6 [56]. NBS1-5 and 7–12 showed reduced serum levels of IgG or IgG subclasses and/or IgA and severely impaired specific antibody responses after vaccination against HBV [57]. NBS6 (p1 in the original report) had hypogammaglobulinaemia and the tetanus and HiB antibody response after immunization was poor or absent [56]. The institutional review board at the Karolinska Institute approved the study.

### RNA isolation and PCR amplification of V_H_3-23-Cγ transcripts

For NBS patients and controls, total RNA was extracted from PBMC using RNeasy RNA purification kits (Qiagen, Germany) and first-strand cDNA synthesis was performed with a CγA primer (5′-GTCCTTGACCAGGCAGCCCAG-3′) using a cDNA synthesis kit (Pharmacia, Uppsala, Sweden). For ATLD patients, total RNA was extracted from B cells using the Ultraspec™ RNA isolation system (Biotex). cDNA was synthesized using a Revers-iT cDNA synthesis kit (Abgene™). The primers used for amplification of V_H_3-23-Cγ transcripts were V_H_3-23 (5′-tctagaGGCTGAGCTGGCTTTTTCTTGTGG-3′) and CγB (5′-cagtcgacAAGA CCGATGGGCCCTTGGTGG-3′). The oligonucleotides contained a restriction site, (underlined, a *Xba* I site in the V_H_3-23 primer and a *Sal* I site in the CγB primer) for directional cloning of the PCR products. Amplification was performed in 35 cycles, each cycle consisting of 94°C 50 sec, 62°C 1 min and 72°C 1 min. High fidelity DNA polymerases *Vent* (New England BioLabs, Hertfordshire, England, GB) or *Pfu* (Fermentas Life Sciences, Burlington, Canada) were used for the amplification.

### PCR amplification of J_H_4 intronic region sequences

PBMCs were isolated by density gradient centrifugation and CD27^+^ cells were separated from PBMCs using CD27 MicroBeads and a MiniMACS Separator (Miltenyi Biotec, CA, USA). The J_H_4 intronic regions were PCR amplified from genomic DNA prepared from PBMC or CD27^+^ PBMC (when fresh blood samples were available) using a FR3 consensus primer and a primer upstream of J_H_5 as described previously [58]. A *Xba* I or a *Sal* I site was added in the J_H_4-FR3 or J_H_5 primer for directional cloning of the PCR products. A long PCR kit (Expand Long Template PCR System Kit, Roche Diagnostics Scandinavia, Bromma, Sweden) was employed to amplify the J_H_4 intronic region in the first set of controls. This system uses an enzyme mixture containing *Taq* DNA polymerase and a proofreading *Pwo* DNA polymerase. The PCR error was estimated to be 2/10000 bp [55]. A high fidelity DNA polymerase *Pfu* (Fermentas Life Sciences) was used in amplifications of the J_H_4 intronic regions from NBS patients and a second set of controls. Genomic DNA from several individuals with known germline *TNFRSF13B* sequences were used to assess the fidelity of this polymerase. Independent PCR products were cloned, and mutations were shown to be introduced at a rate of less than 1/10000 bp in 37 clones analyzed (1 error of the 18027 bp sequenced). There was no significant difference in frequency of mutations and base substitution pattern in the two sets of controls (190 and 425 mutations respectively) and they were thus merged as one group.

### Analysis of V_H_-Cγ and J_H_4 clones

The PCR products were purified and cloned into the Bluescript II KS (+) vector (Stratagene, La Jolla, USA) and transformed into JM 109 competent cells. The resulting clones were screened by PCR amplification (V_H_3-23 and CγB or J_H_4-FR3 and J_H_5) and positive clones were sequenced by an automated fluorescent sequencer in MWG (Ebersberg, Germany) or in Macrogen (Seoul, Korea). The V_H_-Cγ sequence analysis was performed by IMGT/V-QUEST (http://imgt.cines.fr) [59] to align the V_H_-CγB sequences to their closest germline V_H_, D and J_H_ segment counterparts. The immunoglobulin V(D)J junctional sequences were analyzed by the IMGT/JunctionAnalysis tool (http://imgt.cines.fr). The analysis on J_H_4 intronic sequences was performed using the LASERGENE software package (DNASTAR, Madison, WI, USA).

Sequence motifs surrounding mutated bases and trinucleotide targeting were determined as previously described [60], [61]. Briefly, mutated V_H_3-23 or J_H_4 intron sequences were aligned beneath the germline V_H_3-23 or J_H_4 intron region gene and a raw test file of the alignment was created. This file was imported into a Microsoft Excel spreadsheet and computations of the number of each type of nucleotide substitution and the composition of the flanking sequences around these substitutions were performed using macros in Excel (Visual Basic). Computations of percentage differences and χ^2^ analysis were also performed using Excel.

### Online Supplemental Material

Table S1 and S2 show the number of mutations at each base of all trinucleotides in ATLD or NBS patients. Table S3 shows the frequency of mutations within the RGYW/WRCY motifs in V_H_3-23 and J_H_4 intronic sequences in patients and controls. Table S4 shows the nature of base pair substitutions in NBS patients and controls with younger ages (Control II, 1–8 years). Table S5 shows the characteristics of the CDR3 regions in V_H_3-23-Cγ transcripts from ATLD and NBS patients.

## Supporting Information

Table S1 Number of mutations at each base of all trinucleotidesa in ATLD patients
(0.09 MB PDF)

Table S2 Number of mutations at each base of all trinucleotidesa in NBS patients
(0.08 MB PDF)

Table S3 Number of mutations at SHM G/C mutation hotspots in VH3-23 and JH4 intronic sequences
(0.06 MB PDF)

Table S4 Comparison of the nature of base substitutions in the VH3-23
(0.07 MB PDF)

Table S5 Characteristics of the CDR3 regions in VH3-23-Cγ transcripts from ATLD and NBS patients
(0.06 MB PDF)

## References

[1] Chaudhuri J, Alt FW (2004). Class-switch recombination: interplay of transcription, DNA deamination and DNA repair.. Nat Rev Immunol.

[2] Jung D, Giallourakis C, Mostoslavsky R, Alt FW (2006). Mechanism and control of V(D)J recombination at the immunoglobulin heavy chain locus.. Annu Rev Immunol.

[3] Pan-Hammarstrom Q, Zhao Y, Hammarstrom L (2007). Class switch recombination: a comparison between mouse and human.. Adv Immunol.

[4] Muramatsu M, Kinoshita K, Fagarasan S, Yamada S, Shinkai Y (2000). Class switch recombination and hypermutation require activation-induced cytidine deaminase (AID), a potential RNA editing enzyme.. Cell.

[5] Rada C, Williams GT, Nilsen H, Barnes DE, Lindahl T (2002). Immunoglobulin isotype switching is inhibited and somatic hypermutation perturbed in UNG-deficient mice.. Curr Biol.

[6] Di Noia J, Neuberger MS (2002). Altering the pathway of immunoglobulin hypermutation by inhibiting uracil-DNA glycosylase.. Nature.

[7] Bemark M, Sale JE, Kim HJ, Berek C, Cosgrove RA (2000). Somatic hypermutation in the absence of DNA-dependent protein kinase catalytic subunit (DNA-PK(cs)) or recombination-activating gene (RAG)1 activity.. J Exp Med.

[8] Faili A, Aoufouchi S, Gueranger Q, Zober C, Leon A (2002). AID-dependent somatic hypermutation occurs as a DNA single-strand event in the BL2 cell line.. Nat Immunol.

[9] Li Z, Woo CJ, Iglesias-Ussel MD, Ronai D, Scharff MD (2004). The generation of antibody diversity through somatic hypermutation and class switch recombination.. Genes Dev.

[10] Neuberger MS, Di Noia JM, Beale RC, Williams GT, Yang Z (2005). Somatic hypermutation at A.T pairs: polymerase error versus dUTP incorporation.. Nat Rev Immunol.

[11] Imai K, Slupphaug G, Lee WI, Revy P, Nonoyama S (2003). Human uracil-DNA glycosylase deficiency associated with profoundly impaired immunoglobulin class-switch recombination.. Nat Immunol.

[12] Di Noia JM, Williams GT, Chan DT, Buerstedde JM, Baldwin GS (2007). Dependence of antibody gene diversification on uracil excision.. J Exp Med.

[13] Demple B, Herman T, Chen DS (1991). Cloning and expression of APE, the cDNA encoding the major human apurinic endonuclease: definition of a family of DNA repair enzymes.. Proc Natl Acad Sci U S A.

[14] Robson CN, Hickson ID (1991). Isolation of cDNA clones encoding a human apurinic/apyrimidinic endonuclease that corrects DNA repair and mutagenesis defects in E. coli xth (exonuclease III) mutants.. Nucleic Acids Res.

[15] Xanthoudakis S, Smeyne RJ, Wallace JD, Curran T (1996). The redox/DNA repair protein, Ref-1, is essential for early embryonic development in mice.. Proc Natl Acad Sci U S A.

[16] Hadi MZ, Wilson DM (2000). Second human protein with homology to the Escherichia coli abasic endonuclease exonuclease III.. Environ Mol Mutagen.

[17] Ide Y, Tsuchimoto D, Tominaga Y, Nakashima M, Watanabe T (2004). Growth retardation and dyslymphopoiesis accompanied by G2/M arrest in APEX2-null mice.. Blood.

[18] Guikema JE, Linehan EK, Tsuchimoto D, Nakabeppu Y, Strauss PR (2007). APE1- and APE2-dependent DNA breaks in immunoglobulin class switch recombination.. J Exp Med.

[19] Larson ED, Cummings WJ, Bednarski DW, Maizels N (2005). MRE11/RAD50 cleaves DNA in the AID/UNG-dependent pathway of immunoglobulin gene diversification.. Mol Cell.

[20] D'Amours D, Jackson SP (2002). The Mre11 complex: at the crossroads of dna repair and checkpoint signalling.. Nat Rev Mol Cell Biol.

[21] Lavin MF (2007). ATM and the Mre11 complex combine to recognize and signal DNA double-strand breaks.. Oncogene.

[22] Luo G, Yao MS, Bender CF, Mills M, Bladl AR (1999). Disruption of mRad50 causes embryonic stem cell lethality, abnormal embryonic development, and sensitivity to ionizing radiation.. Proc Natl Acad Sci U S A.

[23] Xiao Y, Weaver DT (1997). Conditional gene targeted deletion by Cre recombinase demonstrates the requirement for the double-strand break repair Mre11 protein in murine embryonic stem cells.. Nucleic Acids Res.

[24] Zhu J, Petersen S, Tessarollo L, Nussenzweig A (2001). Targeted disruption of the Nijmegen breakage syndrome gene NBS1 leads to early embryonic lethality in mice.. Curr Biol.

[25] Stewart GS, Maser RS, Stankovic T, Bressan DA, Kaplan MI (1999). The DNA double-strand break repair gene hMRE11 is mutated in individuals with an ataxia-telangiectasia-like disorder.. Cell.

[26] Varon R, Vissinga C, Platzer M, Cerosaletti KM, Chrzanowska KH (1998). Nibrin, a novel DNA double-strand break repair protein, is mutated in Nijmegen breakage syndrome.. Cell.

[27] Lahdesmaki A, Taylor AM, Chrzanowska KH, Pan-Hammarstrom Q (2004). Delineation of the role of the Mre11 complex in class switch recombination.. J Biol Chem.

[28] Pan Q, Petit-Frere C, Lahdesmaki A, Gregorek H, Chrzanowska KH (2002). Alternative end joining during switch recombination in patients with ataxia-telangiectasia.. Eur J Immunol.

[29] Kracker S, Bergmann Y, Demuth I, Frappart PO, Hildebrand G (2005). Nibrin functions in Ig class-switch recombination.. Proc Natl Acad Sci U S A.

[30] Reina-San-Martin B, Nussenzweig MC, Nussenzweig A, Difilippantonio S (2005). Genomic instability, endoreduplication, and diminished Ig class-switch recombination in B cells lacking Nbs1.. Proc Natl Acad Sci U S A.

[31] Yabuki M, Fujii MM, Maizels N (2005). The MRE11-RAD50-NBS1 complex accelerates somatic hypermutation and gene conversion of immunoglobulin variable regions.. Nat Immunol.

[32] Rogozin IB, Pavlov YI, Bebenek K, Matsuda T, Kunkel TA (2001). Somatic mutation hotspots correlate with DNA polymerase eta error spectrum.. Nat Immunol.

[33] Digweed M, Sperling K (2004). Nijmegen breakage syndrome: clinical manifestation of defective response to DNA double-strand breaks.. DNA Repair (Amst).

[34] Taylor AM, Groom A, Byrd PJ (2004). Ataxia-telangiectasia-like disorder (ATLD)-its clinical presentation and molecular basis.. DNA Repair (Amst).

[35] Delbos F, Aoufouchi S, Faili A, Weill JC, Reynaud CA (2007). DNA polymerase eta is the sole contributor of A/T modifications during immunoglobulin gene hypermutation in the mouse.. J Exp Med.

[36] Masuda K, Ouchida R, Hikida M, Kurosaki T, Yokoi M (2007). DNA polymerases eta and theta function in the same genetic pathway to generate mutations at A/T during somatic hypermutation of Ig genes.. J Biol Chem.

[37] Kanno S, Kuzuoka H, Sasao S, Hong Z, Lan L (2007). A novel human AP endonuclease with conserved zinc-finger-like motifs involved in DNA strand break responses.. Embo J.

[38] Xue K, Rada C, Neuberger MS (2006). The in vivo pattern of AID targeting to immunoglobulin switch regions deduced from mutation spectra in msh2-/- ung-/- mice.. J Exp Med.

[39] Xiao Z, Ray M, Jiang C, Clark AB, Rogozin IB (2007). Known components of the immunoglobulin A:T mutational machinery are intact in Burkitt lymphoma cell lines with G:C bias.. Mol Immunol.

[40] Boddy MN, Russell P (2001). DNA replication checkpoint.. Curr Biol.

[41] Olson E, Nievera CJ, Liu E, Lee AY, Chen L (2007). The Mre11 Complex Mediates the S-Phase Checkpoint through an Interaction with Replication Protein A.. Mol Cell Biol.

[42] Takemura H, Rao VA, Sordet O, Furuta T, Miao ZH (2006). Defective Mre11-dependent activation of Chk2 by ataxia telangiectasia mutated in colorectal carcinoma cells in response to replication-dependent DNA double strand breaks.. Journal of Biological Chemistry.

[43] Trenz K, Smith E, Smith S, Costanzo V (2006). ATM and ATR promote Mre11 dependent restart of collapsed replication forks and prevent accumulation of DNA breaks.. EMBO Journal.

[44] Jansen JG, Langerak P, Tsaalbi-Shtylik A, van den Berk P, Jacobs H (2006). Strand-biased defect in C/G transversions in hypermutating immunoglobulin genes in Rev1-deficient mice.. J Exp Med.

[45] Longerich S, Basu U, Alt F, Storb U (2006). AID in somatic hypermutation and class switch recombination.. Curr Opin Immunol.

[46] Maser RS, Zinkel R, Petrini JH (2001). An alternative mode of translation permits production of a variant NBS1 protein from the common Nijmegen breakage syndrome allele.. Nat Genet.

[47] Difilippantonio S, Nussenzweig A (2007). The NBS1-ATM connection revisited.. Cell Cycle.

[48] Fernet M, Gribaa M, Salih MA, Seidahmed MZ, Hall J (2005). Identification and functional consequences of a novel MRE11 mutation affecting 10 Saudi Arabian patients with the ataxia telangiectasia-like disorder.. Hum Mol Genet.

[49] Berkovich E, Monnat RJ, Kastan MB (2007). Roles of ATM and NBS1 in chromatin structure modulation and DNA double-strand break repair.. Nat Cell Biol.

[50] Kitagawa R, Kastan MB (2005). The ATM-dependent DNA damage signaling pathway.. Cold Spring Harb Symp Quant Biol.

[51] Lumsden JM, McCarty T, Petiniot LK, Shen R, Barlow C (2004). Immunoglobulin class switch recombination is impaired in Atm-deficient mice.. J Exp Med.

[52] Pan-Hammarstrom Q, Jones AM, Lahdesmaki A, Zhou W, Gatti RA (2005). Impact of DNA ligase IV on nonhomologous end joining pathways during class switch recombination in human cells.. J Exp Med.

[53] Reina-San-Martin B, Chen HT, Nussenzweig A, Nussenzweig MC (2004). ATM is required for efficient recombination between immunoglobulin switch regions.. J Exp Med.

[54] Yan CT, Boboila C, Souza EK, Franco S, Hickernell TR (2007). IgH class switching and translocations use a robust non-classical end-joining pathway.. Nature.

[55] Pan-Hammarstrom Q, Dai S, Zhao Y, van Dijk-Hard IF, Gatti RA (2003). ATM is not required in somatic hypermutation of VH, but is involved in the introduction of mutations in the switch mu region.. J Immunol.

[56] Gennery AR, Slatter MA, Bhattacharya A, Barge D, Haigh S (2004). The clinical and biological overlap between Nijmegen Breakage Syndrome and Fanconi anemia.. Clin Immunol.

[57] Gregorek H, Chrzanowska KH, Michalkiewicz J, Syczewska M, Madalinski K (2002). Heterogeneity of humoral immune abnormalities in children with Nijmegen breakage syndrome: an 8-year follow-up study in a single centre.. Clin Exp Immunol.

[58] Faili A, Aoufouchi S, Weller S, Vuillier F, Stary A (2004). DNA polymerase eta is involved in hypermutation occurring during immunoglobulin class switch recombination.. J Exp Med.

[59] Giudicelli V, Chaume D, Lefranc MP (2004). IMGT/V-QUEST, an integrated software program for immunoglobulin and T cell receptor V-J and V-D-J rearrangement analysis.. Nucleic Acids Res.

[60] Pan-Hammarstrom Q, Lahdesmaki A, Zhao Y, Du L, Zhao Z (2006). Disparate roles of ATR and ATM in immunoglobulin class switch recombination and somatic hypermutation.. J Exp Med.

[61] Spencer J, Dunn-Walters DK (2005). Hypermutation at A-T base pairs: the A nucleotide replacement spectrum is affected by adjacent nucleotides and there is no reverse complementarity of sequences flanking mutated A and T nucleotides.. J Immunol.

[62] Unniraman S, Schatz DG (2007). Strand-biased spreading of mutations during somatic hypermutation.. Science.

