# Supplementary material for: A Regulatory Role for NBS1 in Strand-Specific Mutagenesis during Somatic Hypermutation
Source: PLoS One. 2008 Jun 25;3(6):e2482. doi: 10.1371/journal.pone.0002482 (PMC2423615; doi:10.1371/journal.pone.0002482)
Supplement: Table S1 — Number of mutations at each base of all trinucleotidesa in ATLD patients (0.09 MB PDF) [file pone.0002482.s001.pdf]

Table S1. Number of mutations at each base of all trinucleotides[a] in ATLD patients

	**ATLD**				**Controls**			
	Position 1	Position 2	Position 3	Total	Position 1	Position 2	Position 3	Total
AAA	2	4	3	9	1	8	3	12
AAC	8	7	5	20	4	6	5	15
AAG	8	13	3	24	6	10	2	18
AAT	9	8	4	21	9	4	6	19
ACA	23	8	12	43	16	13	13	42
ACC	4	7	7	18	4	14	7	25
ACG	1	7	19	27	5	7	16	28
ACT	12	16	1	29	12	12	2	26
AGA	13	9	**13↑**	35	11	8	3	22
AGC	32	74	72	178	35	79	70	184
AGG	12	19	14	45	11	13	8	32
AGT	17	21	8	46	18	21	6	45
ATA	14	5	4	23	18	6	10	34
ATC	10	0	3	13	11	0	6	17
ATG	10	3	5	18	14	0	1	15
ATT	14	5	5	24	14	13	9	36
CAA	2	**14↑**	14	30	6	4	13	23
CAC	18	9	10	37	18	9	20	47
CAG	23	21	41	85	26	21	44	91
CAT	4	10	5	19	5	9	6	20
CCA	11	5	14	30	12	3	9	24
CCC	0	2	0	2	0	1	0	1
CCG	11	3	16	30	11	9	15	35
CCT	9	12	16	37	17	9	14	40
CGA	0	0	**4↑**	4	2	0	0	2
CGC	7	19	14	40	7	15	10	32
CGG	1	1	12	14	1	1	11	13
CGT	2	15	2	19	6	15	2	23
CTA	36	11	**10↓**	57	33	10	23	66
CTC	8	**9↑**	1	18	10	1	3	14
CTG	33	16	19	68	22	15	16	53
CTT	9	6	4	19	7	6	2	15
GAA	4	9	15	28	8	8	12	28
GAC	4	6	5	15	1	1	4	6
**GAG**	8	16	**50↑**	**74↑**	11	8	32	51
GAT	2	2	0	4	0	1	2	3
GCA	25	29	19	73	35	36	15	86
GCC	16	21	6	43	9	22	7	38
GCG	1	3	5	9	2	4	1	7
GCT	79	54	17	150	69	43	14	126
GGA	13	3	4	20	11	5	2	18
GGC	4	19	6	29	3	13	3	19
**GGG**	5	6	15	**26↑**	1	1	9	11
GGT	20	48	17	85	22	50	28	100
GTA	55	17	42	114	54	26	55	135
GTC	4	0	0	4	2	0	2	4
GTG	25	14	18	57	28	13	25	66
GTT	12	2	0	14	10	0	0	10
TAA	0	0	0	0	0	0	0	0
TAC	8	18	18	44	11	21	17	49
**TAG**	18	24	29	**71↓**	28	36	43	107
TAT	16	28	4	48	12	43	5	60
TCA	6	5	9	20	1	3	6	10
TCC	2	1	9	12	2	3	8	13
TCG	0	0	0	0	0	0	0	0
TCT	2	4	8	14	0	8	2	10
TGA	0	6	12	18	2	7	13	22
TGC	6	9	15	30	1	8	22	31
TGG	32	16	35	83	29	22	41	92
TGT	1	12	6	19	0	8	3	11
TTA	2	9	14	25	6	9	12	27
TTC	6	1	6	13	8	2	3	13
TTG	4	3	1	8	6	1	3	10
TTT	2	1	5	8	0	1	2	3
			Sum	2240			Sum	2235

a). Statistical analysis was performed using χ^2 test. Numbers that are significantly different from controls are bolded (p<0.05) and marked with arrows (↑, increased compared to the controls; ↓, decreased compared to the controls). The trinucleotides that are differentially targeted between patient and controls are bolded and bases within a given triplet that are differentially targeted are underlined.
