# Supplementary material for: A Regulatory Role for NBS1 in Strand-Specific Mutagenesis during Somatic Hypermutation
Source: PLoS One. 2008 Jun 25;3(6):e2482. doi: 10.1371/journal.pone.0002482 (PMC2423615; doi:10.1371/journal.pone.0002482)
Supplement: Table S2 — Number of mutations at each base of all trinucleotidesa in NBS patients (0.08 MB PDF) [file pone.0002482.s002.pdf]

**Table S2.** Number of mutations at each base of all trinucleotides[a] in NBS patients

| | NBS | | | | Controls | | | |
|---|---|---|---|---|---|---|---|---|
| | Position 1 | Position 2 | Position 3 | Total | Position 1 | Position 2 | Position 3 | Total |
| AAA | 1 | 8 | 4 | 13 | 1 | 8 | 3 | 12 |
| **AAC** | 6 | 16 | **18↑** | **40↑** | 4 | 6 | 5 | 15 |
| AAG | 3 | 16 | 0 | 19 | 6 | 10 | 2 | 18 |
| AAT | 9 | 8 | 5 | 22 | 9 | 4 | 6 | 19 |
| ACA | 28 | 21 | 11 | 60 | 16 | 13 | 13 | 42 |
| ACC | 4 | 8 | 5 | 17 | 4 | 14 | 7 | 25 |
| ACG | 9 | 5 | 18 | 32 | 5 | 7 | 16 | 28 |
| ACT | 16 | 11 | 2 | 29 | 12 | 12 | 2 | 26 |
| AGA | 13 | 1 | 11 | 25 | 11 | 8 | 3 | 22 |
| AGC | 27 | **136↑** | 80 | 243 | 35 | 79 | 70 | 184 |
| AGG | 14 | 9 | 7 | 30 | 11 | 13 | 8 | 32 |
| AGT | 20 | 37 | 7 | 64 | 18 | 21 | 6 | 45 |
| ATA | 19 | 14 | 7 | 40 | 18 | 6 | 10 | 34 |
| **ATC** | **3↓** | 0 | 5 | **8↓** | 11 | 0 | 6 | 17 |
| ATG | 10 | 1 | 9 | 20 | 14 | 0 | 1 | 15 |
| ATT | 10 | 9 | 9 | 28 | 14 | 13 | 9 | 36 |
| CAA | 3 | 2 | 15 | 20 | 6 | 4 | 13 | 23 |
| CAC | 20 | 13 | 12 | 45 | 18 | 9 | 20 | 47 |
| CAG | 19 | 16 | 64 | 99 | 26 | 21 | 44 | 91 |
| CAT | 3 | 7 | 14 | 24 | 5 | 9 | 6 | 20 |
| CCA | 16 | 2 | 6 | 24 | 12 | 3 | 9 | 24 |
| CCC | 0 | 3 | 0 | 3 | 0 | 1 | 0 | 1 |
| CCG | 6 | 4 | 19 | 29 | 11 | 9 | 15 | 35 |
| CCT | 16 | 7 | 15 | 38 | 17 | 9 | 14 | 40 |
| CGA | 1 | 0 | 0 | 1 | 2 | 0 | 0 | 2 |
| CGC | 5 | 18 | 6 | 29 | 7 | 15 | 10 | 32 |
| CGG | 1 | 0 | 9 | 10 | 1 | 1 | 11 | 13 |
| CGT | 2 | 19 | 2 | 23 | 6 | 15 | 2 | 23 |
| CTA | 44 | 9 | 14 | 67 | 33 | 10 | 23 | 66 |
| CTC | 7 | 1 | 3 | 11 | 10 | 1 | 3 | 14 |
| CTG | 30 | 25 | 20 | 75 | 22 | 15 | 16 | 53 |
| CTT | 7 | 2 | 0 | 9 | 7 | 6 | 2 | 15 |
| GAA | 8 | 9 | **29↑** | 46 | 8 | 8 | 12 | 28 |
| GAC | 0 | 3 | 3 | 6 | 1 | 1 | 4 | 6 |
| GAG | 9 | 9 | 38 | 56 | 11 | 8 | 32 | 51 |
| GAT | 1 | 0 | 2 | 3 | 0 | 1 | 2 | 3 |
| GCA | **60↑** | **21↓** | 13 | 94 | 35 | 36 | 15 | 86 |
| GCC | 11 | 24 | 3 | 38 | 9 | 22 | 7 | 38 |
| GCG | 0 | 1 | 2 | 3 | 2 | 4 | 1 | 7 |
| **GCT** | **107↑** | 63 | 15 | **185↑** | 69 | 43 | 14 | 126 |
| GGA | 9 | 7 | 3 | 19 | 11 | 5 | 2 | 18 |
| GGC | 1 | 9 | 7 | 17 | 3 | 13 | 3 | 19 |
| GGG | 1 | 2 | 9 | 12 | 1 | 1 | 9 | 11 |
| GGT | **11↓** | 57 | 22 | 90 | 22 | 50 | 28 | 100 |
| GTA | 66 | 21 | 48 | 135 | 54 | 26 | 55 | 135 |
| GTC | 2 | 1 | 1 | 4 | 2 | 0 | 2 | 4 |
| GTG | 46 | 16 | 16 | 78 | 28 | 13 | 25 | 66 |
| GTT | 9 | 0 | 0 | 9 | 10 | 0 | 0 | 10 |
| TAA | 0 | 0 | 0 | 0 | 0 | 0 | 0 | 0 |
| TAC | 18 | 25 | 12 | 55 | 11 | 21 | 17 | 49 |
| TAG | 24 | 33 | **81↑** | 138 | 28 | 36 | 43 | 107 |
| **TAT** | 12 | **27↓** | 3 | **42↓** | 12 | 43 | 5 | 60 |
| TCA | 0 | 1 | 8 | 9 | 1 | 3 | 6 | 10 |
| TCC | 3 | 3 | 8 | 14 | 2 | 3 | 8 | 13 |
| TCG | 0 | 0 | 0 | 0 | 0 | 0 | 0 | 0 |
| TCT | 0 | 7 | 5 | 12 | 0 | 8 | 2 | 10 |
| TGA | 0 | 10 | 7 | 17 | 2 | 7 | 13 | 22 |
| TGC | 6 | 15 | 16 | 37 | 1 | 8 | 22 | 31 |
| TGG | 39 | 11 | 50 | 100 | 29 | 22 | 41 | 92 |
| TGT | 0 | 10 | 7 | 17 | 0 | 8 | 3 | 11 |
| TTA | 2 | 10 | 16 | 28 | 6 | 9 | 12 | 27 |
| TTC | 7 | 1 | 2 | 10 | 8 | 2 | 3 | 13 |
| **TTG** | 2 | 0 | 1 | **3↓** | 6 | 1 | 3 | 10 |
| TTT | 0 | 0 | 2 | 2 | 0 | 1 | 2 | 3 |
| | | | Sum | 2476 | | | Sum | 2235 |

a). Statistical analysis was performed using $\chi^2$ test. Numbers that are significantly different from controls are bolded ($p<0.05$) and marked with arrows (↑, increased compared to the controls; ↓, decreased compared to the controls). The trinucleotides that are differentially targeted between patient and controls are bolded and bases within a given triplet that are differentially targeted are underlined.
