# Supplementary material for: A Regulatory Role for NBS1 in Strand-Specific Mutagenesis during Somatic Hypermutation
Source: PLoS One. 2008 Jun 25;3(6):e2482. doi: 10.1371/journal.pone.0002482 (PMC2423615; doi:10.1371/journal.pone.0002482)
Supplement: Table S3 — Number of mutations at SHM G/C mutation hotspots in VH3-23 and JH4 intronic sequences (0.06 MB PDF) [file pone.0002482.s003.pdf]

Table S3. Number of mutations at SHM G/C mutation hotspots in V_H3-23 and J_H4 intronic sequences

	**VH3-23 sequences**			**JH4 intronic sequences**	
	**ATLD** (753 mutations)	**NBS** (829 mutations)	**Control** (750 mutations)	**NBS** (386 mutations)	**Control** (797 mutations)
**<u>RGYW</u>**					
Position 1-A	11 (1.5%)	9 (1.1%)	8 (1.1%)	3 (0.8%))	8 (1.0%)
-G	20 (2.7%)	12 (1.4%)	20 (2.7%)	**9 (2.3%)***	6 (0.8%)
Position 2-G	123 (16.3%)	**190 (22.9%)****	126 (16.8%)	**49 (12.7%)****	59 (7.4%)
Position 3-C	64 (8.5%)	72 (8.7%)	56 (7.5%)	15 (3.9%)	23 (2.9%)
-T	15 (2.0%)	17 (2.1%)	22 (2.9%)	2 (0.5%)	2 (0.3%)
Position 4-A	22 (2.9%)	23 (2.8%)	23 (3.1)	7 (1.8%)	18 (2.3%)
-T	18 (2.4%)	15 (1.8%)	12 (1.6%)	5 (1.3%)	12 (1.5%)
Total	286 (38.0%)	353 (42.6%)	288 (38.4%)	**92 (23.8%)****	132 (16.5%)
**<u>WRCY</u>**					
Position 1-A	18 (2.4%)	11 (1.3%)	11 (1.5%)	8 (2.1%)	12 (1.5%)
-T	10 (1.3%)	**17 (2.1%)****	4 (0.5%)	10 (2.6%)	15 (1.9%)
Position 2-A	6 (0.8%)	13 (1.6)	11 (1.5%)	3 (0.8%)	7 (0.9%)
-G	59 (7.8)	**96 (11.6%)****	55 (7.3%)	27 (7.0%)	35 (4.4%)
Position 3-C	71 (9.4%)	83 (10.0%)	63 (8.4%)	46 (11.9%)	91 (11.4%)
Position 4-C	6 (0.8%)	2 (0.2%)	5 (0.7%)	4 (1.0%)	14 (1.8%)
-T	11 (1.5%)	9 (1.1%)	8 (1.1%)	3 (0.8%)	11 (1.4%)
Total	188 (25.0%)	**234 (28.2%)****	164 (21.9%)	101 (26.2%)	185 (23.2%)
**R<u>G</u>YW and W<u>R</u>CY (duplicates excluded)**					
G mutations total	138 (18.3%)	**200 (24.1%)****	134 (17.9%)	**76 (19.7%)*****	94 (11.8%)
**All sequences**					
G mutations	383 (37.6)	**343 (41.4%)***	267 (35.6)	147 (38.1)**	230 (28.9%)
