# Supplementary material for: A Regulatory Role for NBS1 in Strand-Specific Mutagenesis during Somatic Hypermutation
Source: PLoS One. 2008 Jun 25;3(6):e2482. doi: 10.1371/journal.pone.0002482 (PMC2423615; doi:10.1371/journal.pone.0002482)
Supplement: Table S4 — Comparison of the nature of base substitutions in the VH3-23 (0.07 MB PDF) [file pone.0002482.s004.pdf]

**Table S4.** Comparison of the nature of base substitutions in the $V_H$3-23 genes between NBS patients and the controls (age 1-8 years)

| NBS (% of 829 mutations) | | | | | | |
|---|---|---|---|---|---|---|
| **To:** | T | C | A | G | Total | Corrected |
| **From:** | | | | | | |
| T | / | 6.8 | 3.3 | 3.6 | **13.6↓** * | **16.4↓** * |
| C | 11.2 | / | 3.6 | 7.0 | 21.8 | 22.1 |
| A | 6.4 | 5.1 | / | 11.7 | 23.2 | 27.9 |
| G | 3.7 | **16.6↑** ** | 21.0 | / | **41.4↑** * | **33.6↑** * |
| Total | 21.4 | 28.5 | 27.9 | 22.3 | 100 | |
| G vs. C mutation ratio | | | | **1.9↑** * | | |
| Transversions (at G) | | | | 49.3% (**20.4%↑** **) | | |

| Controls II (% of 808 mutations) | | | | | | |
|---|---|---|---|---|---|---|
| **To:** | T | C | A | G | Total | Corrected |
| **From:** | | | | | | |
| T | / | 7.7 | 4.3 | 5.3 | 17.3 | 20.6 |
| C | 13.2 | / | 3.0 | 8.9 | 25.1 | 25.0 |
| A | 5.9 | 4.7 | / | 11.0 | 21.7 | 25.7 |
| G | 3.2 | 12.0 | 20.7 | / | 35.9 | 28.7 |
| Total | 22.4 | 24.4 | 28.0 | 25.2 | 100 | |
| G vs. C mutation ratio | | | | 1.4 | | |
| Transversions (at G) | | | | 47.4% (15.2%) | | |
