# Supplementary material for: A Regulatory Role for NBS1 in Strand-Specific Mutagenesis during Somatic Hypermutation
Source: PLoS One. 2008 Jun 25;3(6):e2482. doi: 10.1371/journal.pone.0002482 (PMC2423615; doi:10.1371/journal.pone.0002482)
Supplement: Table S5 — Characteristics of the CDR3 regions in VH3-23-Cγ transcripts from ATLD and NBS patients (0.06 MB PDF) [file pone.0002482.s005.pdf]

Table S5. Characteristics of the CDR3 regions in V_H3-23-Cγ transcripts from ATLD and NBS patients

	**Control clones** (n=40)	**ATLD clones** (n=30)	**NBS clones** (n=72)
**Length of CDR3**	14.9 ± 4.2	15.1 ± 3.4	14.1 ± 3.4
**Length of N nucleotides**			
**N1 (V_H-D junction)**	4.8 ± 4.9	6.0 ± 5.1	4.8 ± 4.1
**N2 (D-J_H junction)**	5.5 ± 4.7	6.6 ± 5.5	3.3 ± 4.2*
**P-nucleotide insertion**	15 / 40 (38%)	11 / 30 (37%)	26 / 72 (36%)

* p<0.05, Student's t test
